# Multimodal imaging study of hepatic perivascular epithelioid cell tumors: a case report

**DOI:** 10.3389/fmed.2023.1322048

**Published:** 2023-12-07

**Authors:** Wenbi Yang, Quanlin Sun, Maocai Shang, Song Li, Xiao Hu, Xianwen Hu

**Affiliations:** Department of Nuclear Medicine, Affiliated Hospital of Zunyi Medical University, Zunyi, China

**Keywords:** hepatic, perivascular epithelioid cell tumors, computed tomography, magnetic resonance imaging, positron emission tomography

## Abstract

Hepatic perivascular epithelioid cell tumors (PEComas) are rare interstitial tumors that are often misdiagnosed as hepatocellular carcinomas due to their unique vascular enhancement patterns. Herein, we present a case of a 61-year-old man who was incidentally found to have a lesion in the left medial segment of the liver during a chest computed tomography (CT) examination performed 4 days prior to his presentation for chest discomfort. Imaging revealed solid components with density similar to that of normal liver tissue and areas of low-density adipose tissue within the lesion. The solid components exhibited increased uptake of fluorine-18 fluorodeoxyglucose on positron emission tomography/CT. Magnetic resonance imaging demonstrated areas with unevenly high signal intensity in both T1-weighted imaging (T1WI) in-phase and T2-weighted imaging (T2WI) sequences, while T2WI in the opposite phase displayed areas with unevenly low signal intensity, indicating the presence of fatty components. Contrast-enhanced T1WI displayed a “fast in and fast out” enhancement pattern. These distinct imaging features contribute to the diagnosis of hepatic PEComas and distinguish it from hepatocellular carcinoma.

## Introduction

Perivascular epithelioid cell tumors (PEComas) are a group of mesenchymal tumors with histological and immunohistochemical features characterized by the coexpression of perivascular epithelioid myoid cells and melanocyte markers (1). PEComas are a family of tumors that includes angiomyolipomas, lymphangiomyomatosis, lung clear-cell glycomas, clear-cell myomelanocyte tumors, and clear-cell tumors that occur rarely in the pancreas, rectum, bone, and soft tissue (2). PEComas can occur in various parts of the body, especially in the genitourinary system, which accounts for approximately 40% of cases, followed by lung, pancreas and so on (2). In recent years, reports of hepatic PEComas have increased with increasing attention to the disease (3, 4). The incidence of PEComas in the liver is approximately six times higher in women than in men, with a wide age range and a peak incidence in young and middle-aged individuals (5). Patients typically do not exhibit obvious clinical symptoms, and most of them seek medical attention due to incidental findings from routine physical examinations or discomfort caused by the compression effect of large masses (6). Herein, we present the diagnosis and treatment of a patient with hepatic PEComa, focusing on the imaging features and differential diagnosis, with the hope of raising awareness regarding this rare disease.

## Case presentation

**Figure 1:**
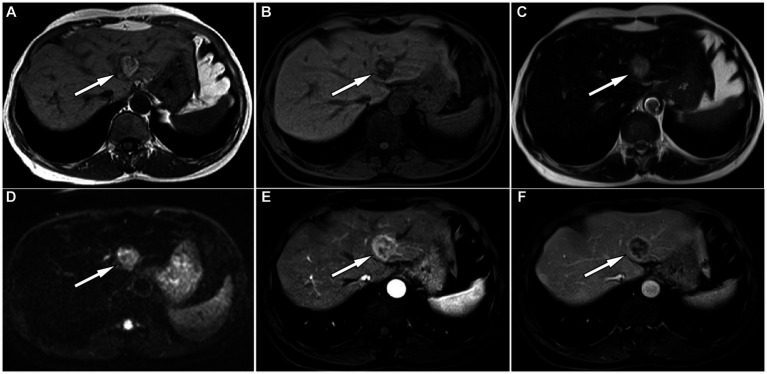
The in-phase T1-weighted imaging (T1WI) of abdominal magnetic resonance imaging (MRI) reveals a high and low mixed signal nodule about 2.0 cm × 1.8 cm in size in the medial segment of the left lobe of liver (**A**, arrow); The reverse phase of T1WI shows that the signal of the high signal part of the original nodules has become low (**B**, arrow), indicating fat composition; The nodule shows slightly high signal on T2-weighted imaging (T2WI) **(C)** and diffusion weighted imaging **(D)**. On contrast-enhanced T1WI, the lesion shows significant enhancement in the arterial phase **(E)** and rapid resolution in the venous phase **(F)**, presenting a typical “fast in and fast out” enhancement pattern.

**Figure 2:**
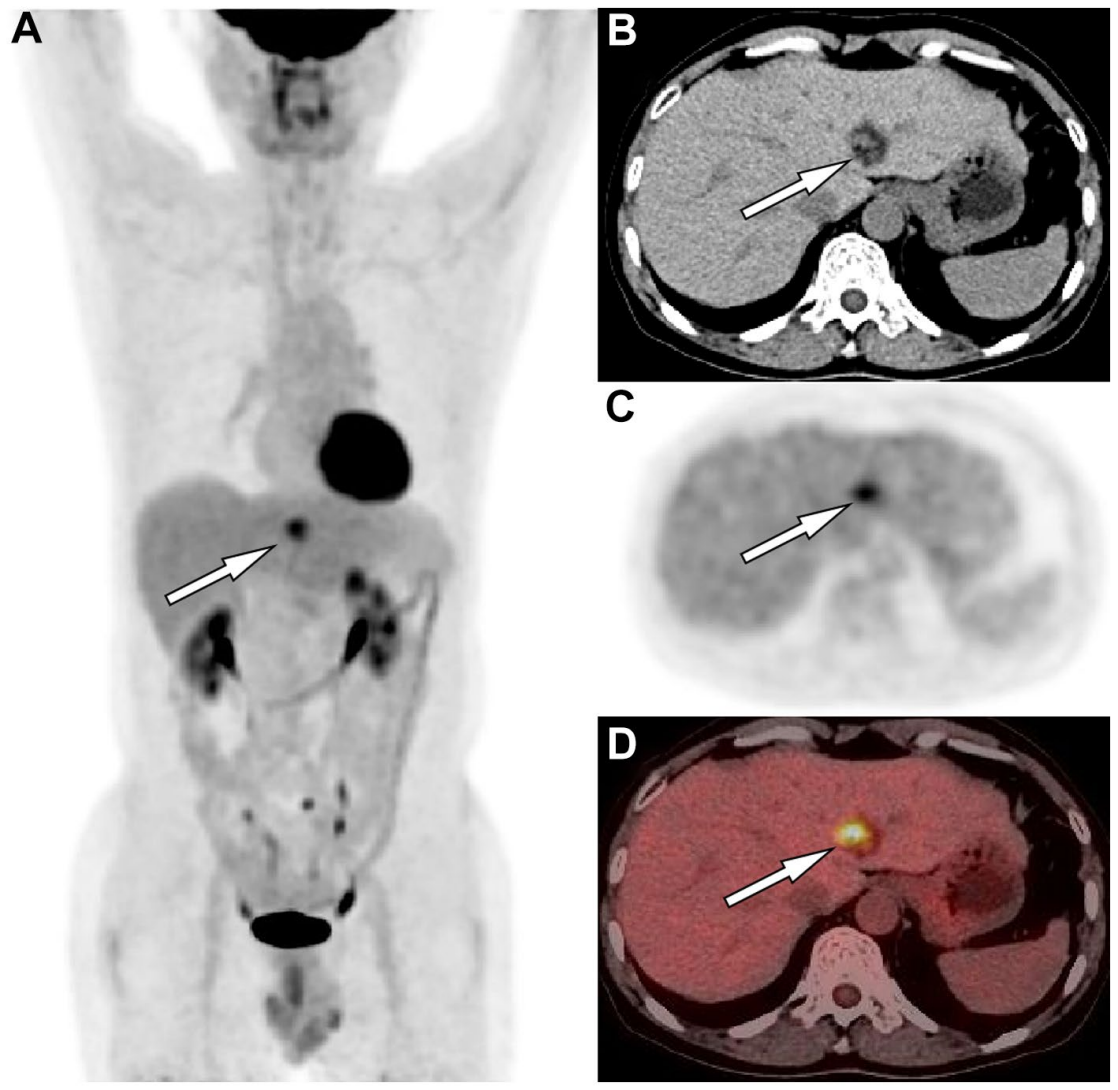
**(A)** The maximum intensity projection of the positron emission tomography (PET)/computed tomography (CT) shows a nodule (arrow) on the left side of the spine with increased uptake of fluorine-18 fluorodeoxyglucose (^18^F-FDG), with a maximum standardized uptake value (SUVmax) of 5.4. Axial images CT **(B)** showed the corresponding nodule at the medial segment of the left lobe of liver, with solid components of equal hepatic parenchymal density and low-density adipose tissue (arrow). Axial PET **(C)** and PET/CT fusion image **(D)** showed an increased ^18^F-FDG uptake of the solid component of the nodule (arrows).

**Figure 3:**
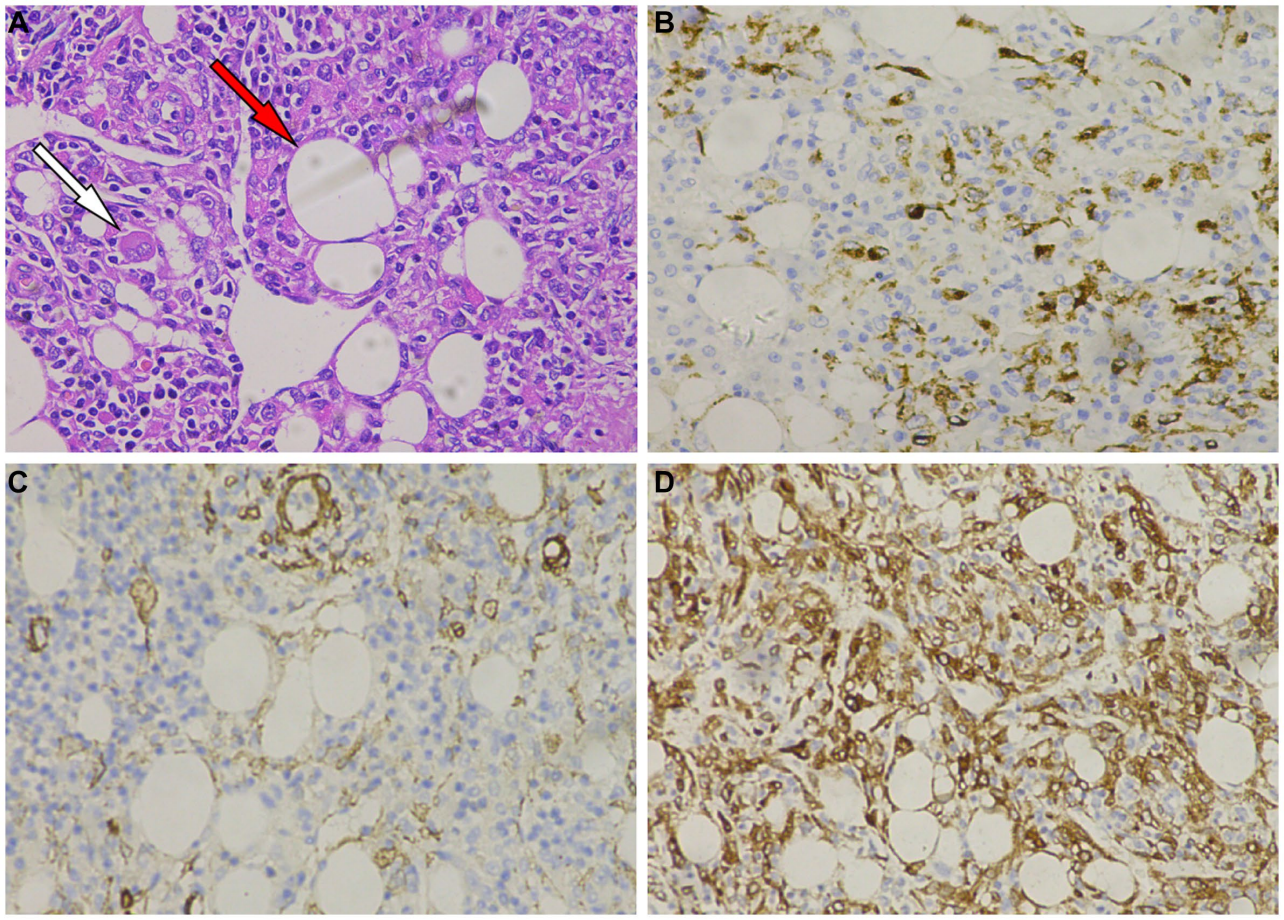
**(A)** Hematoxylin–Eosin staining shows that tumor cells are composed of epithelioid cells, with smooth muscle (white arrow) and adipose tissue (red arrow) visible within the tumor tissue. Immunohistochemistry shows that tumor cells positively express HMB45 **(B)**, melan-A **(C)**, smooth muscle actin (SMA) **(D)**.

A 61-year-old man was admitted to the hospital following the discovery of liver lesions during a chest computed tomography (CT) examination, which had been performed due to chest discomfort 4 days prior. He had no history of hypertension, diabetes, hepatitis, tumors, trauma, or prior surgery. His family also denied any history of tumors or genetic problems; physical examination revealed no positive signs. Laboratory examination showed that except for a slight elevation of total bilirubin (37.5 μmol/L, normal: 5–21 μmol/L) and direct bilirubin (7.9 μmol/L, normal: 0–3.4 μmol/L), other laboratory indicators including blood routine and serum tumor markers of digestive system were within the normal reference value range. CT examination revealed a mixed density nodule approximately 2.0 cm × 1.8 cm in size in the medial segment of the left hepatic lobe. On magnetic resonance imaging (MRI as shown in Figure 1), the lesion presented uneven short T1 and long T2 signals, with a clear “fast in and fast out” appearance on contrast-enhanced scans. Based on these imaging findings, the patient was initially suspected of hepatocellular carcinoma. To determine the best course of treatment, the patient underwent a positron emission tomography (PET)/CT examination (PET/CT imaging presented in Figure 2), which revealed increased fluorine-18 fluorodeoxyglucose (^18^F-FDG) uptake in the lesion, while no significant abnormal radioactive uptake was observed throughout the rest of the body. Subsequently, the patient underwent surgical resection of the lesion under general anesthesia. The excised tumor tissue was sent for histopathological examination; under a microscope, it showed a grayish-red color. The tumor cells as shown in Figure 3 were composed of epithelioid cells rich in transparent cytoplasm and eosinophilic granules. Immunohistochemistry showed that the tumor cells positively expressed HMB45, melan-A, smooth muscle actin (SMA), and calponin. However, they were negative for hepatocyte, microphthalmia-associated transcription factor (MITF), anaplastic lymphoma kinase (ALK), S100, and Ki-67, with a positive index of approximately 10%. Based on the pathological and immunohistochemical results, the patient was diagnosed with hepatic PEComa. The patient was discharged 5 days after receiving antiinflammatory treatment following surgery. To date, the patient has been followed-up for 14 months, and showed no evidence of recurrence.

## Discussion

Hepatic PEComas are usually solitary, with only 5–15% occurring as part of the tuberous sclerosis complex (5). Hepatic PEComas are mainly angiomyolipomas, often characterized by a prominent epithelioid morphology and may lack mature adipose tissue or thick-walled blood vessels, typically presenting as a single phenotype (7). Therefore, it can histomorphologically mimic some hepatocyte-derived tumors, such as hepatocellular adenomas and hepatocellular carcinoma (HCC), leading to an incorrect diagnosis. Patients with liver PEComas do not have specific symptoms and most seek medical attention because of physical examination findings or compression symptoms due to excessive tumor volume. A few patients may experience symptoms such as upper abdominal pain, nausea, indigestion, and loss of appetite. There is also a reported case in literature of one patient presenting with chills and fever (8). In our case, a chest CT examination was performed due to chest discomfort, incidentally revealing a liver lesion.

The preoperative diagnosis of PEComa relies mainly on imaging methods. On CT, PEComas in the liver appear mainly as circular or lobulated mixed-density masses with clear boundaries. The solid components of the tumor appear to be of equal or slightly lower density than the liver parenchyma, and the lesion may contain low-density cystic necrotic areas or fatty components (9). On contrast-enhanced scanning, the enhancement mode of the tumor is related to the proportion of various components in the tumor tissue, mainly presenting as obvious enhancement in the arterial phase accompanied by rapid regression, arterial phase enhancement accompanied by slow regression, arterial phase enhancement accompanied by late continuous enhancement, or uneven enhancement (10). On MRI, the main findings of the tumor are a low signal on T1-weighted imaging (T1WI) and a slightly high signal on T2-weighted imaging (T2WI); in typical cases, large tortuous vascular flow empty signal shadows can be seen inside the visible lesions, which has a certain specificity for the diagnosis of PEComas (11). Moreover, the isoinverse phase of T1WI can determine whether there is fat concomitancy inside the lesion, and cystic necrosis inside the lesion is more clearly visible (12). There are only a few literature reports on PET/CT of liver PEComas, and according to the composition of the epithelioid tissue contained in the tumor, its presentation can range from no ^18^F-FDG uptake to obviously increased ^18^F-FDG uptake (13–15). Our patient showed equal and low mixed densities on CT, with slightly high signal intensity on T1WI in the same phase and low signal intensity in the opposite phase, indicating the presence of fatty components in the lesion. T2WI showed slightly higher signal intensity, and diffusion-weighted imaging showed limited tumor spread. On contrast-enhanced T1WI, the lesion was significantly enhanced in the arterial phase, and rapidly subsided in the portal phase, presenting as a “fast in and fast out” appearance. On PET/CT, the solid components of the tumor showed increased ^18^F-FDG uptake, while the surrounding fat components did not show any uptake. These findings are consistent with the imaging features of hepatic PEComas reported in the aforementioned literature.

The clinical and imaging-based differential diagnosis of hepatic PEComas includes HCC, focal nodular hyperplasia of the liver, hepatic adenoma, liposarcoma, and hemangioma. HCC also presents with a “fast in and fast out” enhancement mode on contrast-enhanced CT and MRI, and cystic necrosis may be associated with large masses. However, patients with HCC usually have a history of hepatitis B or C viral infection and cirrhosis, and serum alpha-fetoprotein is often elevated (16); while hepatic PEComas do not have such characteristics. Focal nodular hyperplasia of the liver appears on CT as an equal-or slightly lower-density mass with a typical star scar in the middle and less fatty components. The contrast-enhanced scan showed that the lesions were significantly enhanced in the arterial stage and gradually decreased in the venous and delayed stages, and the central scar in the delayed stage exhibited delayed enhancement and gradually filled with isodense material compared to the liver parenchyma, thus providing specificity (17). Typical liver adenomas also exhibit “fast in and slow out” in contrast enhancement, and they are more likely to merge with bleeding and are more common in women, with a history of taking contraceptives and steroids (18). Liposarcomas of the liver are relatively rare and contain fat. However, the tumor volume is mostly large, and cystic necrosis is relatively rare. Contrast-Enhanced scanning shows uneven enhancement in all stages, which is different from the “fast in and fast out” feature of liver PEComas (19). Hepatic hemangiomas appear as a slightly low-density mass on CT; the dynamic enhanced scan shows nodular enhancement at the edge of the lesion at the arterial stage, and the lesion gradually fills to the center at the portal and delayed stages, showing a “fast in and slow out” enhancement mode, which distinguishes them from hepatic PEComas (20). The detailed differentiation between hepatic PEComas and other liver lesions is shown in Table 1.

**Table 1:** The clinical features and imaging findings of hepatic PEComas and other liver masses.

Indexs	Clinical features	Epidemicity	Imaging findings			
			CT	MRI	CEM	PET
PEComas	Usually has no symptoms and found by accident	Preference for young and middle-aged women	Low density, typically containing fat	Long T1 and long T2 signals, but fat components showed short T1 and long T2 signals	Most are “fast in and fast out”, and a few are continuous enhancement	Different levels of ^18^F-FDG uptake increased
Hepatocellular carcinoma	Signs of cachexia such as abdominal pain and emaciation; Serum alpha-fetoprotein increased	More common in patients with previous hepatitis or cirrhosis.	Equal or low-density mass, usually accompanied by necrosis	Uneven long T1 and long T2 signals	“fast in and fast out”	Different levels of ^18^F-FDG uptake increased
Focal nodular hyperplasia	Usually has no symptoms and found by accident	More common in middle-aged and elderly patients	Equal or slightly lower density masses	T1WI shows equal or slightly lower signal, T2WI shows equal or slightly higher signal	“Fast in and slow out”, with star shaped scars visible in the center	Moderate uptake of ^18^F-FDG increased, with no uptake in the central stellate scar.
Hepatic adenoma	Usually has no symptoms and found by accident	More common in women with a history of taking birth control pills or steroids	Equal density mass, easily accompanied by high-density bleeding	T1WI shows equal or slightly lower signal, T2WI shows equal or slightly higher signal	“Fast in and slow out”	Mildly increased ^18^F-FDG uptake
Liposarcoma	May manifest as abdominal pain and bloating	More common in adults	Uneven isodense, low-density mass	Both T1WI and T2WI showed uneven signals	Uneven enhancement in all phases	Obviously increased ^18^F-FDG uptake
Hemangioma	Usually has no symptoms and found by accident	More common in middle-aged and elderly people, with more females than males	Uniformly equal or slightly lower density	Long T1 and long T2 signals	More show “Fast in and slow out” while a few show “fast in and fast out”	No or mild increase in ^18^F-FDG uptake

CEM, contrast enhancement model; CT, computed tomography; MRI, magnetic resonance imaging; PET, positron emission tomography; T1WI, T1 weighted imaging; T2WI, T2 weighted imaging.

Histopathology is the gold standard for the diagnosis of PEComas, and epithelial cell morphology and clear eosinophilic cytoplasm can be observed under a microscope with abundant glycogen, premelanin bodies, and half-desmosomes (21). Immunohistochemical staining for PEComas can positively express both muscle cell (SMA, muscle-specific actin, calponin, etc.) and melanocyte (HMB 45, melan A, tyrosinase) markers (1). The tumor cells in this patient were composed of epithelioid cells rich in transparent cytoplasm and eosinophilic granules. Immunohistochemistry showed positive expression of HMB45, melan-A, SMA, and calponin in the tumor cells, which is consistent with the diagnosis of PEComa.

Due to the low incidence of hepatic PEComas, there is no unified standard for related treatment strategies, and surgical resection remains the primary approach for their management. Some studies suggest that for lesions >5 cm, if there is progressive enlargement, presence of clinical symptoms, or an indication of malignant tendency on fine-needle biopsy, more active methods should be used for treatment, and that mTOR inhibitors such as rapamycin have a certain effect on the treatment of malignant PEComas (22, 23). As the biological behavior of most hepatic PEComas is benign, their prognosis is good (24). Our patient underwent surgical resection of the mass without further treatment, was followed up for 14 months, and is still healthy and alive, which is consistent with literature reports.

## Conclusion

Hepatic PEComas are rare interstitial tumors that are often misdiagnosed as hepatocellular carcinoma on imaging due to their unique rich blood supply enhancement pattern. However, the imaging findings did possess a certain degree of specificity. The presence of solid components with density similar to that of hepatic parenchyma and adjacent low-density adipose tissue within the lesion, along with increased uptake of ^18^F-FDG, is characteristic. Contrast enhancement typically exhibits a “fast in and fast out” pattern. In cases of hepatic lesions with these features, considering the possibility of PEComas is crucial for an accurate diagnosis.

## Data availability statement

The original contributions presented in the study are included in the article/supplementary material, further inquiries can be directed to the corresponding author.

## Ethics statement

Written informed consent was obtained from the individual(s) for the publication of any potentially identifiable images or data included in this article.

## Author contributions

WY: Conceptualization, Formal analysis, Methodology, Writing – original draft. QS: Investigation, Project administration, Supervision, Writing – original draft. MS: Conceptualization, Data curation, Project administration, Writing – original draft. SL: Data curation, Software, Validation, Writing – original draft. XiaoH: Data curation, Project administration, Software, Writing – original draft. XianH: Conceptualization, Formal analysis, Methodology, Resources, Writing – review & editing.

## References

[1] Folpe AL Kwiatkowski DJ. Perivascular epithelioid cell neoplasms: pathology and pathogenesis. Hum Pathol. (2010) 41:1–15. doi: 10.1016/j.humpath.2009.05.011 19604538

[2] Martignoni G Pea M Reghellin D Zamboni G Bonetti F. PEComas: the past, the present and the future. Virchows Arch. (2008) 452:119–32. doi: 10.1007/S00428-007-0509-1, PMID: 18080139 PMC2234444

[3] Gao X Tang H Wang J Yao Q Wang H Wang Y. Specific imaging features indicate the clinical features of patients with hepatic perivascular epithelioid cell tumor by comparative analysis of CT and ultrasound imaging. Front Oncol. (2022) 12:908189. doi: 10.3389/Fonc.2022.908189 36324566 PMC9618795

[4] Matrood S Görg C Safai Zadeh E Alhyari A. Hepatic perivascular epithelioid cell tumor (PEComa): contrast-enhanced ultrasound (CEUS) characteristics-a case report and literature review. Clin J Gastroenterol. (2023) 16:444–9. doi: 10.1007/S12328-023-01779-W 36964879 PMC10199841

[5] Son HJ Kang DW Kim JH Han HY Lee MK. Hepatic perivascular epithelioid cell tumor (PEComa): a case report with a review of literatures. Clin Mol Hepatol. (2017) 23:80–6. doi: 10.3350/Cmh.2016.0034 28288506 PMC5381835

[6] Ameurtesse H Chbani L Bennani A Toughrai I Beggui N Kamaoui I. Primary perivascular epithelioid cell tumor of the liver: new case report and literature review. Diagn Pathol. (2014) 9:149. doi: 10.1186/1746-1596-9-149, PMID: 25034830 PMC4223599

[7] Jia J Luo J Pan CG Ge G Feng M Zou B. Single-center experience in the diagnosis and treatment of hepatic perivascular epithelioid cell neoplasm. J Clin Transl Hepatol. (2022) 10:72–9. doi: 10.14218/Jcth.2020.00170, PMID: 35233375 PMC8845148

[8] Song Z Xu F Dai C. Chills and fever as the first presentation of hepatic perivascular epithelioid cell tumor. Hepatobiliary Surg Nutr. (2019) 8:436–8. doi: 10.21037/Hbsn.2019.07.03, PMID: 31489326 PMC6700025

[9] O'malley ME Chawla TP Lavelle LP Cleary S Fischer S. Primary perivascular epithelioid cell tumors of the liver: CT/MRI findings and clinical outcomes. Abdom Radiol. (2017) 42:1705–12. doi: 10.1007/S00261-017-1074-Y 28246920

[10] Nie P Wu J Wang H Zhou R Sun L Chen J. Primary hepatic perivascular epithelioid cell tumors: imaging findings with histopathological correlation. Cancer Imaging. (2019) 19:32. doi: 10.1186/S40644-019-0212-X, PMID: 31171030 PMC6555711

[11] Tan Y Xiao EH. Hepatic perivascular epithelioid cell tumor (PEComa): dynamic CT, MRI, ultrasonography, and pathologic features—analysis of 7 cases and review of the literature. Abdom Imaging. (2012) 37:781–7. doi: 10.1007/S00261-012-9850-1 22278345

[12] Cardoso H Silva M Vilas-Boas F Cunha R Lopes J Maia JC. Hepatic perivascular epithelioid tumor (PEcoma). a case report. Clin Res Hepatol Gastroenterol. (2017) 41:E43–6. doi: 10.1016/J.Clinre.2017.02.003 28359636

[13] Wang X Wang J Cheng X Li F Huo L. Hepatic angiomyolipoma having FDG uptake at the similar level of the normal liver parenchyma. Clin Nucl Med. (2019) 44:599–601. doi: 10.1097/Rlu.0000000000002551, PMID: 31135516

[14] Wang S Xia H Liu X Liu Y Lou C. Hepatic epithelioid angiomyolipoma mimicking hepatocellular carcinoma on MR and (18)F-FDG PET/CT imaging: a case report and literature review. Hell J Nucl Med. (2022) 25:205–9. doi: 10.1967/S002449912480 35913867

[15] Zhang Y Li B Hou J Yu H Shi H. Hepatic epithelioid angiomyolipoma and 18f-FDG PET/CT. Clin Nucl Med. (2018) 43:422–4. doi: 10.1097/Rlu.0000000000002048, PMID: 29578870

[16] Forner A Reig M Bruix J. Hepatocellular carcinoma. Lancet. (2018) 391:1301–14. doi: 10.1016/S0140-6736(18)30010-2 29307467

[17] Legout JD Bolan CW Bowman AW Caserta MP Chen FK Cox KL. Focal nodular hyperplasia and focal nodular hyperplasia-like lesions. Radiographics. (2022) 42:1043–61. doi: 10.1148/Rg.210156, PMID: 35687520

[18] Aziz H Brown ZJ Eskander MF Aquina CT Baghdadi A Kamel IR. A scoping review of the classification, diagnosis, and management of hepatic adenomas. J Gastrointest Surg. (2022) 26:965–78. doi: 10.1007/S11605-022-05246-8 35083725

[19] Terunuma Y Takahashi K Doi M Shimomura O Miyazaki Y Furuya K. Primary pleomorphic liposarcoma of the liver: a case report and literature review. Surg Case Rep. (2021) 7:244. doi: 10.1186/S40792-021-01322-4, PMID: 34797454 PMC8603980

[20] Liu Z Yi L Chen J Li R Liang K Chen X. Comparison of the clinical and MRI features of patients with hepatic hemangioma, epithelioid hemangioendothelioma, or angiosarcoma. BMC Med Imaging. (2020) 20:71. doi: 10.1186/S12880-020-00465-4, PMID: 32600273 PMC7322860

[21] Tan Y Zhang H Xiao EH. Perivascular epithelioid cell tumour: dynamic CT, MRI and clinicopathological characteristics--analysis of 32 cases and review of the literature. Clin Radiol. (2013) 68:555–61. doi: 10.1016/J.Crad.2012.10.021 23245276

[22] Nguyen TT Gorman B Shields D Goodman Z. Malignant hepatic angiomyolipoma: report of a case and review of literature. Am J Surg Pathol. (2008) 32:793–8. doi: 10.1097/Pas.0b013e3181607349 18391749

[23] Dickson MA Schwartz GK Antonescu CR Kwiatkowski DJ Malinowska IA. Extrarenal perivascular epithelioid cell tumors (Pecomas) respond to Mtor inhibition: clinical and molecular correlates. Int J Cancer. (2013) 132:1711–7. doi: 10.1002/Ijc.27800, PMID: 22927055 PMC3558545

[24] Perán Fernández C De Paco Navaro Á Castañer Ramón-Llin J Bertelli Puche J Sánchez Espinosa A. Perivascular epithelioid cell tumor (PEComa) of the liver. An extremely rare diagnosis. Rev Esp Enferm Dig. (2023) 117:348–9. doi: 10.17235/Reed.2023.9558/2023 37232182

